# Combined PET Imaging and Diffusion-Weighted Imaging of Intermediate and High-Risk Primary Prostate Carcinomas with Simultaneous [^18^F] Choline PET/MRI

**DOI:** 10.1371/journal.pone.0101571

**Published:** 2014-07-17

**Authors:** Axel Wetter, Felix Nensa, Marcus Schenck, Philipp Heusch, Thorsten Pöppel, Andreas Bockisch, Michael Forsting, Thomas W. Schlosser, Thomas C. Lauenstein, James Nagarajah

**Affiliations:** 1 Department of Diagnostic and Interventional Radiology and Neuroradiology, University Hospital Essen, Essen, Germany; 2 Department of Urology and Paediatric Urology, University Hospital Essen, Essen, Germany; 3 University Dusseldorf, Medical Faculty, Department of Diagnostic and Interventional Radiology, Dusseldorf, Germany; 4 Department of Nuclear Medicine, University Hospital Essen, Essen, Germany; Banner Alzheimer’s Institute, United States of America

## Abstract

**Purpose:**

To characterize intermediate and high-risk prostate carcinomas with measurements of standardized uptake values (SUVs) and apparent diffusion coefficient (ADC) values by means of simultaneous [^18^F] choline PET/MRI.

**Materials and Methods:**

35 patients with primary prostate cancer underwent simultaneous [^18^F] choline PET/MRI. From these, 21 patients with an intermediate and high risk constellation who were not under ongoing hormonal therapy were included. Altogether 32 tumor lesions with a focal uptake of [^18^F] choline could be identified. Average ADC values (ADC_aver_) minimum ADC values (ADC_min_) as well as maximum and mean SUVs (SUV_max_, SUV_mean_) of tumor lesions were assessed with volume-of-interest (VOI) and Region-of-interest (ROI) measurements. As a reference, also ADC_aver,_ ADC_min_ and SUV_max_ and SUV_mean_ of non-tumorous prostate tissue were measured. Statistical analysis comprised calculation of descriptive parameters and calculation of Pearson’s product moment correlations between ADC values and SUVs of tumor lesions.

**Results:**

Mean ADC_aver_ and ADC_min_ of tumor lesions were 0.94±0.22×10^−3 ^mm^2^/s and 0.65±0.21×10^−3 ^mm^2^/s, respectively. Mean SUV_max_ and SUV_mean_ of tumor lesions were 6.3±2.3 and 2.6±0.8, respectively. These values were in each case significantly different from the reference values (p<0.001). There was no significant correlation between the measured SUVs and ADC values (SUV_max_ vs. ADC_aver_: R = −0.24, p = 0.179; SUV_max_ vs. ADC_min_: R = −0.03, p = 0.877; SUV_mean_ vs. ADC_aver_: R = −0.27, p = 0.136; SUV_mean_ vs. ADC_min_: R = −0.08, p = 0.679).

**Conclusion:**

Both SUVs and ADC values differ significantly between tumor lesions and healthy tissue. However, there is no significant correlation between these two parameters. This might be explained by the fact that SUVs and ADC values characterize different parts of tumor biology.

## Introduction

Prostate cancer is a common disease of the elder male patient in western countries [1]. Diagnosis of prostate cancer is usually confirmed with ultrasound guided biopsies if a patient reveals a rising PSA level. Especially tumors with an intermediate and high-risk constellation are of clinical interest, as these tumors tend to have a more aggressive growth pattern and show a higher risk of PSA-failure after therapy in comparison to low risk cancers [2]. Over the past years, multiparametric MR imaging of the prostate has evolved a powerful tool for the diagnosis of prostate cancer [3]. In multiparametric MR imaging of the prostate, T2-weighted imaging, diffusion-weighted imaging (DWI), dynamic contrast-enhanced imaging (DCE) and MR spectroscopic imaging (MRSI) are combined to improve diagnostic accuracy. Especially DWI has been investigated to a large extent, however with varying results [4]. So far, value of DWI is limited by a lack of standardization and reproducibility [5]. DWI allows quantitative measurements by calculating the apparent diffusion coefficient (ADC). In prostate cancer the ADC typically has lower values than in benign lesions of the prostate [4]. There are reports that ADC values inversely correlate with the grade of malignancy of prostate cancers in such a way that tumors with high Gleason scores have lower ADC values than tumors with low Gleason scores [6]. Hence, ADC values could be used to identify clinically significant more aggressive prostate cancers. In oncologic imaging, the ADC can be used as an indicator of therapeutic response during chemotherapy, as it has been reported that ADC values tend to rise under ongoing treatment [7], [8]. This can be explained with a disintegration and decrease of tumor cells leading to an alleviation of water diffusion. The successful application of DWI for evaluation of treatment response has been shown for a variety of different tumors, including liver metastases, gynecological malignancies and head and neck cancer [9].

PET/CT imaging of prostate cancer with radio-labelled choline is controversial discussed in the literature because choline is a quite unspecific tracer showing a considerable overlap in uptake between malignant and benign prostate lesions like prostatic hyperplasia [10]. However, some authors report a benefit of choline PET for the detection of prostate cancer. Particularly for the detection and characterization of intermediate and high risk prostate carcinomas choline PET seems to be advantageous [11]. Furthermore, choline PET appears to allow a monitoring of hormonal treatment by indicating a decrease in choline metabolism as a response to hormonal therapy [12]. With the introduction of simultaneous PET/MRI it is possible to combine DWI and PET imaging with high resolution T2-weighted prostate images [13]. Hence, it is possible to perform quantitative measurements of prostate carcinomas on a molecular and metabolic level during a single examination. The goal of this study was to analyze intermediate and high-risk prostate carcinomas with simultaneous [^18^F] choline PET/MRI in order to find a possible correlation between DWI and PET.

## Materials and Methods

### Ethics Statement

The study was approved by the local ethics committee of the University Duisburg-Essen, Germany, and informed written consent was obtained from every patient.

### Patients

From April 2012 to December 2013, 35 patients with biopsy proven primary prostate cancer were examined with simultaneous [^18^F] choline PET/MRI. We included patients for analysis with an intermediate and high risk constellation (i.e. PSA-level 10 mg/dL or higher and/or Gleason sum score of 7 or higher) who were not under ongoing hormonal or radiation therapy or had been treated with radiation therapy and had tumor lesions with a focal uptake of [^18^F] choline. Applying these inclusion criteria, we identified 21 patients for analysis. 14 patients were excluded from analysis; 4 patients did not exhibit a high risk constellation, 5 patients were under ongoing hormonal therapy, 3 patients had a radiation therapy before, one patient displayed a diffuse choline uptake and in one patient the DWI dataset could not be analyzed due to pronounced susceptibility artifacts. In 12 patients, detailed histological reports of TRUS-guided biopsies were used as the standard of reference. In the remaining 9 patients, detailed histological reports of radical prostatectomy specimens prepared as defined by the standards of step section histology with three to five mm slices were used as the standard of reference.

### PET/MRI procedure

PET/MRI scans started 159.7±40.7 minutes after injection of 324.2±46.7 [^18^F] choline and were conducted on a Magnetom Biograph mMR Scanner (Siemens Healthcare, Erlangen, Germany) allowing simultaneous operation of the MR scan and PET scan. PET/MRI scans comprised a pelvic scan with 1 bed position. PET acquisition time was 20 minutes. The mean examination time was 35 minutes. Reconstruction of the PET data was executed using an AWOSEM algorithm with 3 iterations and 21 subsets (512×512 matrix, zoom 1, slice thickness according to the MRI parameter). A post reconstruction Gaussian filter with 5.0 mm fullwidth at half maximum was applied. Attenuation correction of the PET data was accomplished using a four-compartment-model attenuation map (µ-map) according to the method described by Martinez-Möller et al. [14]. A protocol of the employed MR sequences is provided in Table 1.

**Table 1:** Sequence parameters.

Sequence	TR (ms)	TE (ms)	FoV (mm)	Slice thickness (mm)	Matrix	B-values (s/mm^2^)
TIRM coronal	3110	56	380	5	448	
T2 FSE axial	4311	114	400	7	512	
T1 FSE axial	445	9.6	400	7	512	
T1 vibe fs axial	4.41	2.15	420	3	512	
T1 fs axial	808	11	200	2	512	
T2 FSE axial	4320	101	200	3	320	
T2 FSE coronal	4000	101	200	3	320	
DWI	9600	93	260	3.6	160	0, 800, 1000

Abbreviations: VIBE: Volume Interpolated Breathhold Examination, TIRM: Turbo Inversion Recovery, SE: spin echo, FSE: fast spin echo, FoV: field of view, DWI: diffusion weighted imaging, fs: fat saturated.

### SUV and ADC measurements

Measurements of SUVs and ADC values were performed on OsiriX and Syngo TrueD workstations. PET images, T2-weighted images, diffusion-weighted images and ADC maps were imported and synchronized. Maximum and mean SUVs as well as average and minimum ADC values were measured in tumor bearing lesions using a volume-of-interest and region-of-interest method. After correlation with histological results, tumor lesions were identified in T2-weighted MR images and PET images as discrete, focal or ill-defined or invasive/space occupying hypo-intense lesions with a focal uptake of [^18^F] choline. Measurements of SUVs were performed on accordant lesions on fused image data sets. Volumes of interest (VOI) were positioned within a lesion and SUVmax and SUVmean within a 3D isocontour at 50% of SUVmax were determined. ADC values were measured on ADC maps calculated from diffusion-weighted images at a b-value of 1000 s/mm^2^ with region-of-interests drawn closely around the tumor lesion. Furthermore, maximum and mean SUVs and average ADC values were measured in visually classified non tumorous prostate areas. Non tumorous prostate areas were identified after correlation with histological results as regions without focal or diffuse choline uptake and without MR signs of malignancy like above-mentioned. To ensure a concurrent analysis of SUVs and ADC values, PET images and ADC maps were opened in parallel and VOIs as well as ROIs were placed in identical regions. All measurements were performed by one board-certified radiologist and one board-certified nuclear medicine physician.

### Statistical Analysis

The means of SUV_max_ and SUV_mean_ as well as ADC_aver_ and ADC_min_ of tumor lesions and reference areas were calculated and compared with the Wilcoxon signed-rank test. Correlations between SUV_max_, SUV_mean_ and ADC_aver_ and ADC_min_ of tumor lesions and reference lesions were tested with Pearson product-moment correlation. A post-hoc sample size estimation was calculated using power analysis.

All statistical calculations were performed using the R-software environment for statistical computing (R Foundation for Statistical Computing, Vienna, Austria).

## Results

### Patient characteristics and number of lesions

**Figure 1:**
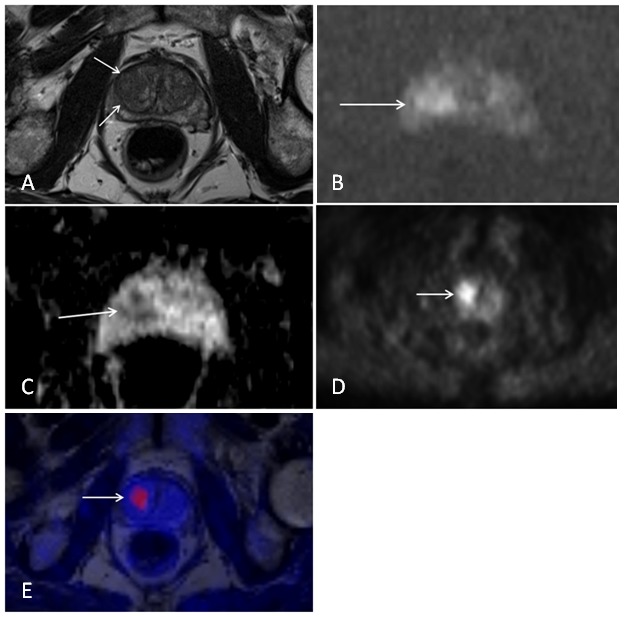
Patient with a biopsy proven prostate cancer of the right transitional zone (Gleason score 3+4 = 7). Images from simultaneous [^18^F] choline PET/MRI. A) T2-weighted image showing an ill-defined hypo-intense lesion of the right transitional zone. B) Diffusion-weighted image at a b-value of 1000 displaying a hyper-intense signal within the lesion indicating restricted water diffusion. C) ADC map with a corresponding hypo-intensity of the lesion. D) PET image showing a focal uptake of (18F) choline of the lesion. E) Fused MRI/PET image.

**Figure 2:**
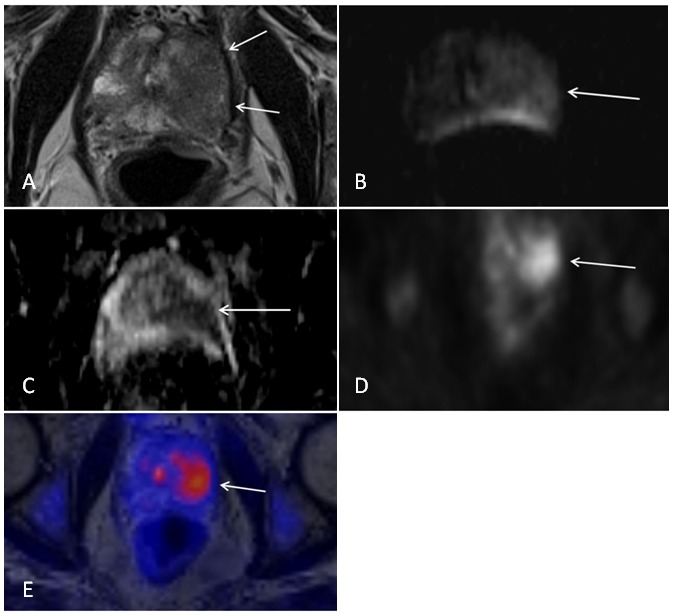
Patient with a biopsy proven prostate cancer (Gleason score 3+4 = 7) of the left peripheral and transitional zone. Images from simultaneous [^18^F] choline PET/MRI. A) T2-weighted image displaying a large hypo-intense lesion of the left peripheral and transitional zone. B) Diffusion-weighted image (b = 1000) showing a hyper-intense signal of the lesion. C) Corresponding ADC map with a hypo-intense delineation of the lesion. D) PET image indicating of focal choline uptake of the lesion. E) Fused MRI/PET image.

Twenty-one patients with primary biopsy proven prostate cancer met the inclusion criteria of our study. Median age was 69 years with a range from 49 years to 80 years. Mean PSA-level was 25.7±23.1 mg/dL. Median Gleason sum score was 7 with a range from 5 to 10. Altogether 32 tumor lesions with a focal uptake of [^18^F] choline were analyzed in 21 patients with biopsy-proven prostate cancer and an intermediate and high risk constellation. 23 lesions were located in the peripheral zone, 5 lesions were located in the transitional zone and 4 lesions were mixed region tumors. Illustrative examples of T2-weighted images, diffusion-weighted images and PET images of prostate tumors are provided in figure 1 and figure 2.

### Maximum and mean standardized uptake values in tumor lesions and reference areas

SUV_max_ and SUV_mean_ in tumors had a mean value of 6.3±2.3 and 3.7±1.2, respectively. Mean values of SUV_max_ and SUV_mean_ in reference areas were 2.6±0.8 and 1.8±0.7, respectively. The difference between mean values of SUV_max_ and SUV_mean_ of tumors and reference areas was statistically significant (p<0.001).

### ADC_aver_ and ADC_min_ values in tumor lesions and reference areas

Mean ADC_aver_ and ADC_min_ values of tumors were 0.94±0.22×10^−3 ^mm^2^/s and 0.65±0.21×10^−3 ^mm^2^/s, respectively. ADC_aver_ values of reference regions were 1.49±0.21×10^−3 ^mm^2^/s. The difference of ADC values in tumors and reference lesions was statistically significant (p<0.001).

### Pearson product-moment correlations (Fig. 3, 4, 5 and 6)

**Figure 3:**
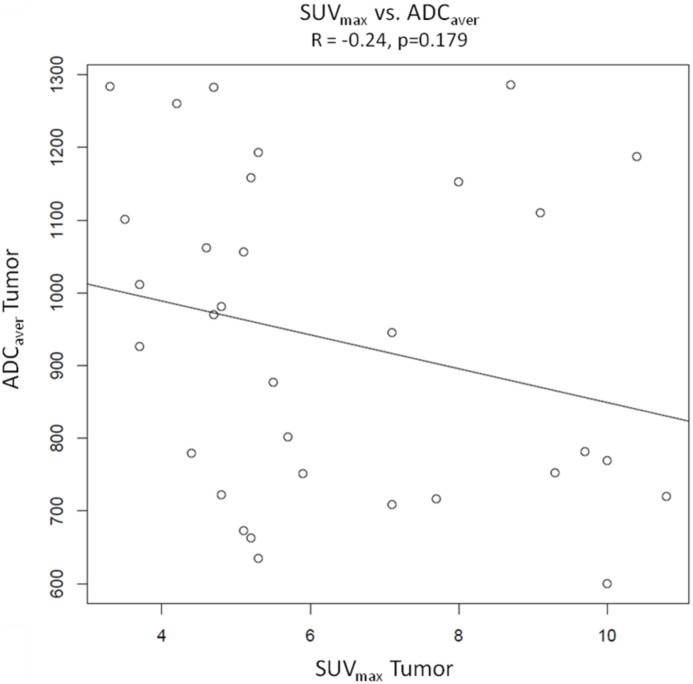
Graphical illustration of Pearson’s product moment correlation of SUV_max_ vs. ADC _aver_. The scatter plot demonstrates a weak negative correlation between SUV_max_ and ADC_aver_ (R = −0.24), which is statistically not significant (p = 0.179).

**Figure 4:**
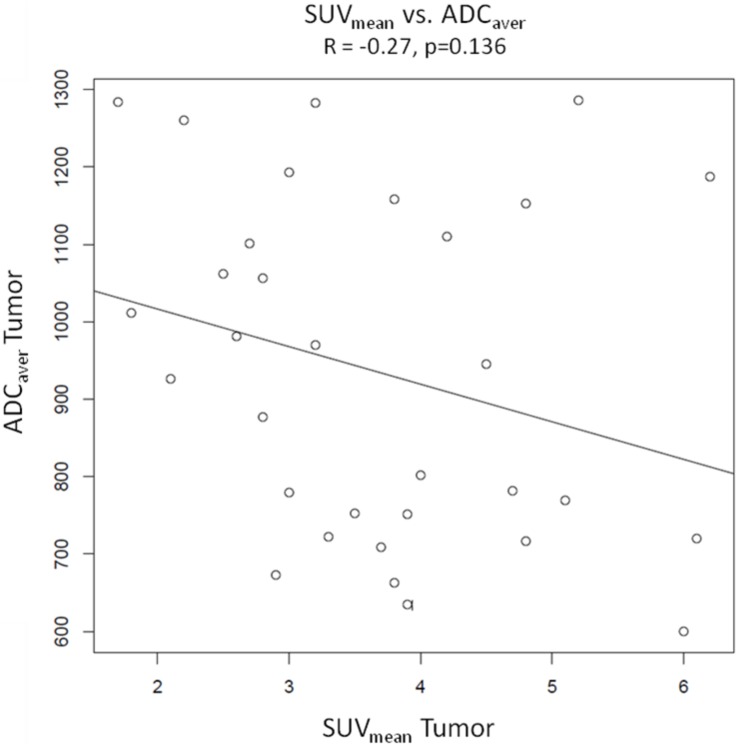
Pearson’s product moment correlation of SUV_mean_ vs. ADC _aver_. The scatter plot indicates a weak negative correlation between SUV_max_ and ADC_aver_ (R = −0.27), which is statistically not significant (p = 0.136).

**Figure 5:**
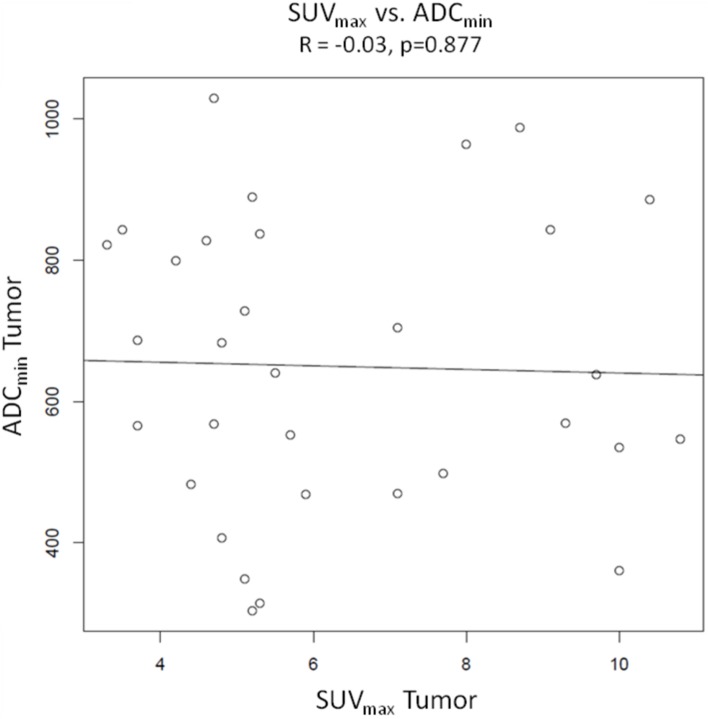
Pearson’s product moment correlation of SUV_max_ vs. ADC_min_. There is no correlation between the two parameters (R = −0.03, p = 0.877).

**Figure 6:**
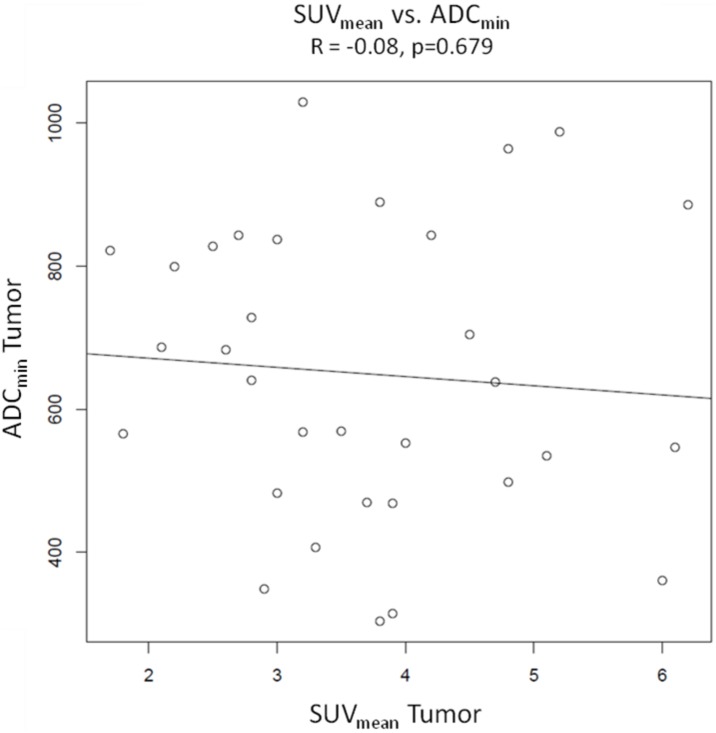
Pearson’s product moment correlation of SUV_mean_ vs. ADC_min_. There is no correlation between the two parameters (R = −0.08, p = 0.679).

Pearson product-moment correlations were calculated from values of SUV_max_ and SUV_mean_ of tumors and ADC_aver_ and ADC_min_ of tumors. There were no significant correlations between the different variables. The Correlation coefficient was R = −0.24 with a p value of 0.179 for SUV_max_ of tumors vs. ADC_aver_ values of tumors and R = −0.27 (p = 0.136) for SUV_mean_ of tumors vs. ADC_aver_ values of tumors. For SUV_max_ vs. ADC_min_ and SUV_mean_ vs. ADC_min_ the correlation coefficients were R = −0.03 (p = 0.877) and −0.08 (p = 0.679), respectively. Sample size calculation indicated that the correlations between SUV_max_ vs. ADC_aver_ values and SUV_mean_ vs. ADC_aver_ values probably would have become significant with 133 patients and 105 patients, respectively.

## Discussion

The introduction of simultaneous PET/MRI made it possible to perform functional and metabolic studies of the prostate during a single examination. The new method of choline PET/MRI has been evaluated in some studies and proved to be feasible for routine use [15], [16]. Some authors describe significant inverse correlations between SUVs and ADC values in different tumor entities. Recently, Rakheja et al. found statistically significant inverse correlations between maximum SUVs and minimum ADC values and mean SUVs and mean ADC values in 69 [^18^F] FDG avid malignant lesions derived from simultaneous FDG PET/MRI [17]. Nakajo et al. report of significant inverse correlations of maximum SUVs derived from FDG PET/CT and ADC values in squamous cell carcinoma of the head and neck region [18]. However, there are also reports revealing that SUVs and ADC values do not inversely correlate significantly, as for example shown by Varoquaux et al. in a study about head and neck squamous cell carcinoma [19]. In addition, a study by Choi et al demonstrated a positive correlation of the ADC ratio and FDG uptake in head and neck cancer [20].

In our study, we examined a potential inverse correlation between SUVs and ADC values of tumor lesions in primary prostate cancer. Our results indicate no significant correlation between the two parameters. The explanation of this result might be that SUVs and ADC values derived from [^18^F] choline PET/MRI in primary prostate cancer reflect different parts of tumor pathophysiology. Whereas choline PET measures the metabolic activity of prostate tumors indicated by their choline uptake over time, DWI indicates restricted water diffusion in prostate tumors, which is mainly caused by increased cellularity and reduced extracellular space. Hence, DWI and choline PET characterize different parts of tumor tissue and biology and therefore an inverse correlation between the two parameters cannot necessarily be expected. The increase of choline uptake in cancers is due to a deregulation of choline metabolism on a cellular basis caused by an upregulation of choline enzyme expression, which can be of a different degree [21]. A key enzyme of choline metabolism is choline kinase, which is frequently overexpressed in tumor cells [22]. In this respect, Contractor et al. demonstrated a strong relationship between choline kinase alpha expression and [^11^C] choline uptake in prostate tumor samples [23]. The same study found no correlation between the Ki67 index and standardized uptake values of [^11^C] choline. Regarding Ki67 and diffusion weighted imaging in prostate cancer, a negative correlation between ADC values and Ki67 expression has been reported [24], [25]. Heijmen et al [26] demonstrated a strong inverse correlation between ADC values and Ki67 expression in liver metastases from colorectal cancer. Furthermore, they found a strong positive correlation between Ki67 expression and nuclear density, which was in turn negatively correlated with ADC values. The observation that choline kinase expression is independent from Ki67 expression in prostate tumors [23] might be one explanation for the absent correlation between ADC values and SUVs in our study.

While the mean values of SUVs and ADC values of tumors and reference areas were significantly different, we observed quite a high variability of SUVs as well as ADC values in tumor lesions. For ADC values, the phenomenon of a significant variability of tumor ADC values is documented in the literature [27]. Additionally, there are reports about a substantial overlap of ADC values of tumor lesions and benign lesions like prostatitis [28]. The same holds true for PET imaging with [^11^C] choline or [^18^F] choline where a substantial overlap was reported between benign lesions like benign prostatic hyperplasia and tumor lesions [13], [29]. Therefore, we would not recommend a threshold for the detection of focal prostate lesions either for ADC values or SUVs and image interpretation should be based on qualitative characteristics.

There are some reports that choline PET imaging could be used for treatment monitoring in patients with prostate cancer [12], [30], [31]. Challapalli et al. [12] report of metabolic changes of the prostate under neoadjuvant androgen deprivation and radiotherapy with concurrent androgen deprivation in terms of a significant decrease of SUVs. In an experimental setting, Schwarzenböck et al. [30] showed that uptake of [^11^C] choline decreased under docetaxel therapy. The value of treatment monitoring has also been reported for diffusion weighted imaging, both for response evaluation to radiotherapy [32] and to androgen deprivation in a preclinical setting [33] with documentation of a rising ADC value after therapy. As both choline PET imaging and DWI are potentially suited to monitor hormonal deprivation therapies as well as radiation therapies, one could think about a scenario where both modalities are combined in one examination like in simultaneous PET/MRI. The simultaneous acquisition of functional and metabolic data by means of integrated PET/MRI requires only one single examination and thereby alleviates the workflow in tumor characterization as compared to the sequential technique of PET/CT followed by MRI. The fact that SUVs and ADC values do not correlate in primary prostate cancers might offer a complementary value of both methods in such a way that both methods reflect different types of response to therapy and therefore might allow a more differentiated evaluation of treatment success.

A clear limitation of our study is the relatively small study group with only 21 patients. Simultaneous PET/MRI is a new method and therefore we were not able to provide a larger patient group. Regarding the calculated correlations, sample size estimation indicated that only a much larger patient group would probably have resulted in significant correlations. However, even in case of significance, the calculated correlations would remain weak.

In conclusion we present our first data on analysis and comparison of SUVs and ADC values in intermediate and high risk primary prostate cancers by means of simultaneous [^18^F] choline PET/MRI. We found no significant correlation of both parameters indicating that SUVs and ADC values derived from simultaneous PET/MRI might be independent biomarkers of primary prostate cancer.
